# Meaning-Making Coping Among Cancer Patients in Sweden and South Korea: A Comparative Perspective

**DOI:** 10.1007/s10943-017-0383-3

**Published:** 2017-03-24

**Authors:** Fereshteh Ahmadi, Jisung Park, Kyung Mee Kim, Nader Ahmadi

**Affiliations:** 1 Department of Social Work and Psychology, Faculty of Health and Occupational Studies, University of Gävle, Gävle, Sweden; 2 Retirement Research Center at Samsung Life Insurance, 25th Flr., Samsung Electronics Bldg., 11 Seocho-daero 74-gil, Seocho-gu, Seoul, 06620 Korea; 3 Department of Social Welfare, Soongsil University, Seoul, Korea

**Keywords:** Meaning-making coping, Cancer, Spirituality, Religion, Sociocultural perspective

## Abstract

The present study compared meaning-making coping among cancer patients in Sweden and South Korea, with a focus on the sociocultural context. Semi-structured interviews were conducted with 51 Swedes and 33 Koreans. The results showed significant differences between the two countries as well as similarities in existential, spiritual, and religious coping. For example, Swedes primarily used meaning-making coping as a means of meditation or relaxation, whereas Koreans relied on coping with prayer and using healthy foods as a means to survive. The present study confirms the significance of investigating cultural context when we explore the use of meaning-making coping among people who have experienced cancer.

## Introduction

Studies on the role of spirituality, religion, and existential issues in health have traditionally overlooked non-religious populations. Such an imbalance has been addressed in recent years, when there has been a greater focus on secular society and the issues secular people face (Ahmadi 2006, 2015; Ahmadi and Ahmadi 2013 La Cour and Hvidit 2010; Moylan et al. 2015).

While many researchers have examined the role of religion and spirituality in health care (Cobb et al. 2012; Koenig et al. 2012; Padela and Curlin 2012; Phelps et al. 2009; Tarakeshwar et al. 2006), studies of religious and spiritual coping have not paid a great deal of attention to patients who follow their own individual spirituality rather than institutional religiosity, or who express no interest in religiosity or spirituality. To take this group into consideration and fill the gap in the research, an international project was established.

The purpose of the international project is to explore meaning-making coping (that is, existential, spiritual, and religious coping) in secular societies among people who have experienced cancer. The international project is expected to help explain the influence that culture has on the use of meaning-making coping methods among people in the countries included. The first studies, both qualitative and quantitative, were conducted in Sweden. After the results were published in Sweden (from the qualitative study in 2006 and the quantitative study in 2015), a series of qualitative studies have been conducted in South Korea, China, and Turkey to date. Japanese researchers have also decided to join the international project, and they plan to conduct a qualitative study this year.

The rationale for choosing these countries is that a considerable part of their respective populations do not identify themselves as religious (with exception of Turkey). Existential questions (what some researchers have called “secular spirituality”) are strong among these populations (Ahmadi 2006; Reed 2007: 5–6). As for East Asian countries, Reed (2007) used the Standard Measures of Secularization and found that the populations of East Asia were highly secularized. According to Reed, about half of East Asian respondents were found to believe in an unseen spiritual world that may affect events in the world that they see around them.

Researchers in South Korea, China, and Turkey have conducted qualitative semi-structured interviews with persons who have survived or suffered from cancer. In each country, the interview questions were basically the same, although some questions were modified to better suit the country’s sociocultural context. We chose cancer patients for this international project, because a cancer diagnosis may be one of the most severe life stressors and may be associated with the possibility of death and with fear (Musick et al. 1998). As Johnson and Spilka (1991) mentioned, while dealing with the stressors and tension of cancer, there is a chance that cancer patients will actively turn to meaning-making coping (existential, spiritual, or religious), which may affect their life to a great extent (Ahmadi 2006).

The present study compared meaning-making coping among cancer patients in Sweden and South Korea. Two research questions were posed in the study: (1) what are the similarities in and differences between meaning-making coping in Sweden and South Korea? and (2) is the sociocultural context associated with meaning-making coping in the two countries?

## Theoretical Framework

### Culture, Religion, and Coping

When individuals face life challenges and attempt to understand what is happening in their lives, they go through a process of coping (Ganzevoort 1998). Pargament (1997) defined coping as the search for meaning in times of stress. Coping seems to be multi-layered and contextual, because it concerns an interaction between the individual and the situation (Lazarus and Folkman 1984; Pargament 1997).

Researchers have investigated the positive impact of religious coping on the life of people who have experienced serious illnesses such as cancer (Ano and Vasconcelles 2005; Rana et al. 2015; Tarakeshwar et al. 2006). However, discussions of religious coping should consider situations that weave religion and coping together. Individuals tend to find religion more accessible when religion is already a major part of their orientation system, that is, the way in which culture influences a person’s life. In such cases, people who face life crises tend to rely on religion, because it is more accessible than other resources found in the sociocultural context of their society.

In cultures that have large non-religious resources and where religion plays a smaller role in individuals’ everyday lives, religion also plays a minor role in the coping process. The likelihood that someone will “turn to religion in coping” is closely related to the position that religion has in the individual’s culture. If religion becomes a more integral part of the individual’s orientation system, it will play a more important role in coping, and vice versa (see, e.g., Ahmadi 2006; Ellison 1991; Ferraro and Koch 1994; Kesselring et al. 1986; Neighbors et al. 1983; Wicks 1990).

Ahmadi (2006) conducted a qualitative study in Sweden among people who experienced cancer and found that the nature of certain coping methods was neither religious nor spiritual. These coping methods were not associated with anything transcendent (God or spiritual power), but had much more to do with a connection to nature, to self, and to others—a connection that seemed to be secular.

In the previous studies, existential coping strategies (e.g., connection to nature) were often regarded as either religious or spiritual, even when they had nothing to do with any religion or spirituality (Ahmadi 2006, 2015). When people cope with their life crises, they try to find a source that can help them fill the vacuum created by these life crises. The endeavors of people who attempted an existential search for meaning were interpreted as spiritual in the existing theoretical framework. To some extent, Pargament’s Sanctification theory (Pargament and Mahoney 2005) may have influenced such a tendency. In other words, previous researchers have not been responsive enough to what people were actually saying about their diverse coping strategies (religious, spiritual, or existential).

As Salander (2015) reminded us when discussing Frankl’s (1959) early thoughts on the construct of meaning, meaning-making has a cognitive origin, not a divine one. If we wish to be more responsive to people’s existential coping strategies, it may be more appropriate to look at secular theories such as attribution theory (Fölsterling 2001) or object relations theory (Winnicott 1971).

Salander (2012) explained Winnicott’s object relations theory, writing that people “play with reality” from early childhood to death (p. 39), and mentioned an intermediate area (Winnicott 1971), which refers to a mental space between the internal and external world. When people face unexpected life challenges such as a cancer, they may try to elaborate what is happening in their external reality and interpret its meaning in their internal world.

Taking these issues into consideration, Ahmadi (2015) suggested the term “meaning-making coping,” which could prevent any misunderstandings about non-religious coping methods. In the present study, the term meaning-making coping is thus used to address the whole spectrum of religious, spiritual, and existential coping methods used among cancer patients in the different countries involved.

It appears as though people in Sweden, South Korea, China, Turkey, and Japan have a greater spiritual than a religious orientation. For this reason, it would be interesting to determine what differences or similarities these people have in their choice of meaning-making coping methods. Put another way, if a society’s cultural and social characteristics fundamentally influence the role that religion and spirituality play in coping, it would be interesting to know whether people in countries where religion does not play an important role in everyday life turn to religion or choose religion-oriented spirituality or non-religious spirituality when they face a crisis.

### The Constructs of Religion and Spirituality

The present study utilizes Ahmadi’s definition of religion and spirituality, which was partly based on the definition suggested by Jenkins and Pargament (1995: 52). According to Ahmadi (2006: 72), religion is:

A search for significance that unfolds within a traditional sacred context. It is then related to an organized system of belief and practice relating to a sacred source that includes individual and institutional expressions, serves a variety of purposes, and may play potentially helpful and/or harmful roles in people’s lives.

In this definition, as in Zinnbauer’s (Zinnbauer and Pargament 2005: 35–36), the search for significance is confined to the framework of a traditional sacred context and reflects modern attitudes that object to traditional authority and institutional expressions of belief. Unlike Pargament’s definition (Zinnbauer et al. 1999: 909), Ahmadi’s is sensitive to how people self-identify in cultural settings dominated more by spirituality than religiosity; in this way, the definition enables communication with the general public.

Although spirituality is harder to define and compartmentalize than religion is, Ahmadi’s (2006) study required a working definition, so one was proposed that was based partly on the work of Ahmadi and partly on that of Jenkins and Pargament (1995: 52–53). Ahmadi’s (2006: 71–72) definition of spirituality is as follows:

A search for connectedness with a sacred source that is related or not related to God or any religious holy sources. Spirituality involves efforts to consider metaphysical or transcendent aspects of everyday life as they relate to forces, transcendent and otherwise. Hence, spirituality encompasses religion as well as many beliefs and practices from outside the normally defined religious sphere.

The construct of spirituality defined in the present study is in accordance with the definition of Puchalski et al. (2009). They defined spirituality as “that aspect of humanity which refers to the way individuals seek and express meaning and purpose and the way they experience their connectedness to the moment, to self, to others, to nature, and to the significant or sacred” (Moylan et al. 2015: 222).

Accordingly, in the present project, spirituality refers to that which can be experienced without faith, myths, legends, founding super-personalities, or superstition and which can be practiced both inside and outside a religious context. In a secular and rationally organized society such as Sweden, most people are considered irreligious. People socialized in such an environment may find it difficult to accept the above-mentioned aspects of conventional religions as the truth.

Consequently, our definition focused on the type of spirituality that one experiences inside and outside a religious context. However, studies conducted in Sweden on people who had suffered from cancer (Ahmadi 2006; Ahmadi and Ahmadi 2013; Ahmadi 2015) have shown that other types of coping strategies, such as those connected to nature, were prevalent, and such strategies can hardly be regarded as religious or spiritual. Salander (2015) touched on this issue by relying (without using coping terms) on a more psychological perspective to try to understand the ways in which people deal with existential predicaments. One can define this type of coping strategy as existential, with reference to how people search for meaning without making any connection to religion or religious symbols. The present study uses meaning-making coping to address all religious, existential, and spiritual coping methods.

### The Study in Sweden

Ahmadi (2006) conducted the first qualitative study in Sweden. A total of 51 persons (19 men and 32 women) between 25 and 83 years of age participated in the interviews. To analyze the material, the researchers arranged the interviewees into three groups based on their answers to questions about their outlook on God, religion, and their philosophy of life. The three groups were the “Atheists,” who did not believe in God or spiritual beings; the “Theists,” who believed in a personal God; and the “Non-theists,” who did not believe in a personal God, but did believe in spiritual beings. No agnostics were identified among the interviewees. Ten interviewees were categorized as atheists, 15 as theists, and 32 as non-theists. The location of the cancerous tumors varied among participants.

The results of the study in Sweden showed new coping methods among cancer patients, such as the following: (1) sanctification of nature; (2) a spiritual connection with oneself; (3) positive solitude; (4) altruism; and (5) spiritual music. Coping using sanctification of nature means that the informants sought spirituality in nature to manage their disease, whereas a spiritual connection with oneself means that the informants did not seek a spiritual relationship with God or another sacred being, but instead sought a spiritual relationship with themselves. Positive solitude was a way to reach a higher state of serenity and spirituality by being alone, thinking and reflecting on the situation and overcoming the stressful situation. Altruism or empathy as a coping method among Swedish cancer patients did not refer to managing their mental problems and stress by getting help or offering their services to others or engaging with others, as we see in some religious coping methods. The motivation here was not religious, but an abstract transcendent feeling, a feeling of unity, of being one with all existence. Finally, some informants indicated that spiritual music was an important coping method used to overcome the stress and anxiety caused by cancer. It should be noted that the results of a quantitative study conducted among cancer patients in Sweden (Ahmadi 2015) confirmed the results of the qualitative study (Ahmadi 2006).

### The Study in South Korea

In the Korean study, the goal was to study which of these meaning-making methods—which have been ignored in previous studies in the research field of religious and spiritual coping—were used by the cancer patients in South Korea, and also to discover what new meaning-making methods could be found. The purpose was to develop our theory about the importance of culture in coping.

A total of 33 people participated in the qualitative interviews in Seoul and its suburban areas. The sample included 17 males and 16 females, between 25 and 75 years of age, and those who identified themselves as non-religious, spiritual or religious (Protestant, Catholic, and Buddhist—the three largest religious groups in South Korea). The participants who completed the interviews ranged from having early to severe stages of various types of cancer, and their social status also varied. Eleven people stated that they had no religion, although that does not mean they were distant from spiritual or existential coping. For more details on the study in South Korea, see Ahmadi et al. (2016).

The results of the South Korean study revealed the following coping methods: (1) belief in the healing power of nature; (2) mind–body connection; (3) relying on transcendent power; and (4) finding oneself in relationships with others. Belief in the healing power of nature means participants believed that, while they were suffering from cancer, being close to nature would help to heal them, both their mind and their body. This type of belief had two main themes: mountains as healers and the healing power of natural foods. Some participants appeared to believe strongly that mountains had healing power, and almost all of them mentioned natural foods when discussing the healing power of nature. These participants believed that such foods were a form of anticancer medicine.

When participants were asked whether they had ever considered why they had contracted cancer, they reported believing that stress and an uneasy mind were contributing factors. They felt that a negative and complex state of mind could be part of the reason why the body malfunctions, thus showing their belief in a mind–body connection. Several participants used the Korean expression *ah*-*dung*-*bah*-*dung*, which means that they had put all their efforts into surviving and achieving material success. The notion of mind–body connection was categorized into two sub-themes: peaceful mental attitude and positive life perspective. Relying on transcendent power means that participants felt a need to rely on some omnipotent being when they were being operated upon. Regardless of whether they were religious or not, participants found consolation from saying prayers in that desperate situation. Finally, several participants stated that the most helpful coping resource against cancer was “people.” Cancer patients may experience feelings of solitude during their struggle with cancer. The participants found that they were actually surrounded by friends, neighbors, or fellow patients, all of whom were sincere and supportive. Through experiencing cancer, Korean participants found themselves in relationships with other people.

## Methods

Interview questions for the study in South Korea were mainly constructed on the basis of the results from the Swedish study (Ahmadi 2015). A semi-structured open-ended interview guide for all participants was used (Patton 2002, 2015). The interview questions focused on four areas based on the research questions: (1) situational factors and current life style; (2) coping behaviors; (3) religiosity; and (iv) religious and spiritual coping. The situational factors and current life style included questions about disease stage, time of diagnosis, the type of cancer, and the location of the tumor. Coping behaviors included the questions such as “what thoughts did you have when you were first diagnosed of cancer?”, “how did you accept the reality that you had cancer?” Religiosity included questions such as “do you believe in God?”, “how did you think of God or life giving force when you had cancer?” Religious and spiritual coping included the questions such as “how did your faith or religious practices changed while you were struggling with cancer?” The semi-structured open-ended interview guide that was used in Sweden was translated into English and Korean in order to conduct interviews with Korean participants. Some questions and terms were modified to make them better suited to the South Korean culture.

We chose a convenient sample both in Swedish and South Korean study. In Sweden, a total of 51 participants were recruited by contacting the Swedish Cancer Society and several other organizations related to cancer. These organizations provided the list of participants who willingly volunteered to be interviewed. In South Korea, 33 participants were recruited in Seoul and its suburban areas. Medical social workers at the oncology center at Severance Hospital of Yonsei University and Samsung Seoul Hospital and a senior nurse at the cancer ward at Kyung Hee University Medical Center introduced their patients who agreed to join the interview. Upon receiving the contact information, the interviewers made a phone call to each participant and set the interview schedule. Participants were also recruited through the interviewers’ acquaintances and contacted in the same manner. Before each interview started, participants read the informed consent carefully and signed on it. Participants were told that their participation was voluntary and they could end the interview at any time. Also, it was mentioned that all identifying information would be kept confidential and that they would be identified with a fictitious name. The Uppsala Ethical Vetting Board (equivalent to an institutional review board) in Sweden approved the international project as a whole.

In order to analyze the data, two researchers in Sweden and two researchers in South Korea conducted the coding and thematizing and created groups of different themes for each country. Then they condensed the codes into categories and developed tentative coding schemes in English to enable a comparative analysis. The research team analyzed each category of themes and finalized the coding schemes by adding, excluding, and combining categories. The four authors discussed cultural aspects of the Korean participants’ coping strategies and tested the validity of the coding schemes, with additional advice from a senior researcher who was teaching qualitative research methods in South Korea. To establish confirmability—which is one of the four evaluative criteria suggested by Guba and Lincoln (1994)—the authors considered and discussed their own biases and stereotypes. The authors tried to minimize the impact of their biases and stereotypes and to maintain their objectivity throughout the process of comparative analysis.

Credibility is a determination of whether the research provides a realistic portrayal of participants and other data sources (Erlandson et al. 1993). In this study, credibility is addressed through triangulation, consulting with colleagues, and attaining transferability by using thick description of verbatim statements (Lincoln and Guba 1985). First, triangulation of analysts was supported by using multiple researchers across Sweden and South Korea (Patton 2002, 2015). Two researchers in Sweden interviewed with cancer patients in person, developed codes to analyze the Swedish data, coded the transcripts, developed themes, and wrote the findings. In the same manner, two researchers in South Korea interviewed with cancer patients, developed codes to analyze the data, coded the transcripts, and developed themes. When the themes for comparative analyses were emerged, four researchers closely communicated in order to discuss the research findings together. The four researchers compared the findings from the previous studies of Sweden and South Korea, and discussed the similarities and differences in meaning-making coping in the two countries. In this way, multiple perspectives were discussed among four researchers in the process of data collection, data analyses and syntheses, and writing the research findings.

Second, the researchers asked knowledgeable colleagues to check the validity of data collection and data analyses procedures as Erlandson et al. (1993) advised. Consulting with these colleagues also helped four researchers share diverse perspectives on the research findings and reminded them of the significance of cultural context.

Finally, transferability can be described as the extent to which the research findings can be applied in other contexts or with other respondents (Lincoln and Guba 1985). In qualitative research, the description of the interrelationship and intricacies of the context being studied in great details can support transferability (Erlandson et al. 1993). In the present study, the researchers used thick descriptions of verbatim statements to strengthen transferability. By providing in-depth account of what each participant really said, researchers were able to conduct comparative analyses of the two different cultures.

## Results

Sweden is one of the Scandinavian countries and is located in northern Europe, whereas South Korea is one of the East Asian countries and is located at a great distance from and to the east of Sweden. Concerning their geographical separation, it might seem that the two countries would have little in common. Interestingly, however, the Swedish and Korean participants in our study showed considerable similarities as well as distinctive aspects with respect to the meaning-making coping methods they employed to deal with a life crisis—cancer. In the following, we will compare the results obtained from the Swedish and the Korean studies.

### Finding Coping Resources in Nature

#### Seeking Comfort in Nature

Seeking comfort and tranquility in nature was found to be one of the most powerful coping methods both in Sweden and in South Korea. Ninety-seven percent of Sweden is uninhabited, 69% of Sweden consists of forests, and slightly more than 80% of Swedes live within five kilometers of a national park, nature reserve or other nature conservation site (see https://sweden.se/nature/swedes-love-nature). South Korea also has plenty of mountains and rivers nationwide. In South Korea, about 80% of the land consists of mountains. About 10% is mountains that are 1000 m and higher, and more than 40% are from 200 to 500 m high. The eight provinces of South Korea may be characterized by the mountain chains running either vertically (from north to south) or horizontally (from east to west). Considering such environmental conditions, it is not surprising that participants from the two countries tended to feel intimate with nature and to find comfort in it while struggling with cancer.

One 53-year-old Swedish woman expressed this as follows:

I rise with nature so to speak…I guess I carry that with me as a heritage from my parents, but after cancer, it happens more often that I go to the forest, outside, deliberately to find peace and tranquility. This has helped me a lot in finding my mental balance.

Like the Swedish woman, one 65-year-old Korean woman expressed her appreciation of nature as follows:

When I walk to the forest, the air is so fresh. I would listen to what other women say about the world outside and do exercises with a fitness machine…I used to cry when relying on and looking at the mountain. I feel relieved when I throw out my mind to the mountain. I almost live in the forest these days.

Both Swedish and Korean participants found that nature helped them cope with difficulties. Staying closer to nature, they experienced peace and comfort and found that nature functioned as a remedy for their stressors.

#### Different Perspectives on Nature

While Swedish and Korean participants showed similar patterns of meaning-making coping with nature, the two groups were different in the following respects. In Sweden, three patterns of coping were found in relation to nature. Swedish participants sought spirituality in nature, found tranquility through nature, and experienced a spiritual connection with nature when they were managing their disease. Nature was considered to be a sacred place, much like a church. Judging from the interviews, it would seem that the Swedish interviewees are more acquainted with the construct of spirituality than the Korean people are. Korean participants appreciated the value of nature as a remedy for their cancer, but they hardly expressed their thoughts by using the term spirituality (pronounced *young*-*sung* in Korean). When Korean participants were struggling with cancer, they found that nature was an effective healer to cure the disease, rather than “sanctifying” nature and seeking spirituality in nature. This is well expressed by a 58-year-old Korean man who survived pancreatic cancer. He explained how much he appreciated the healing power of the mountain and pine trees in fighting cancer:

I went to Gayang Mountain every weekend after completing chemotherapy. I believe Gayang Mountain saved my life. Although I was weak physically, I tried to go there and walk slowly. My wife and I rested under the pine trees, listened to the radio and ate the food we brought. We stayed there for about 5–6 h every single day. I liked to be there around 2 pm when the scent of pine tree resin was the strongest. I think pine trees have the power to heal cancer. Gayang Mountain contains great amounts of anion because it borders the west sea. Anion is very good for cancer patients.

Although both Sweden and South Korea are non-religious countries, people’s understanding of spirituality turned out to be different. Swedish people seemed to have a tendency to sanctify nature and to express their thoughts on nature in terms of spirituality (Ahmadi 2006: 133). In contrast, Korean people rarely mentioned the term “spiritual” or “spirituality” when they explained how they valued nature. Korean participants liked to stay closer and be part of nature not only because they found comfort in it, but also because they believed nature would cure their disease (Ahmadi et al. 2016).

Such differences become more apparent when we look at the coping behaviors of Korean participants. In contrast to Swedish participants, most Korean participants suggested one or more kinds of healthy foods from nature as a way to fight cancer. They believed strongly that these foods (mostly vegetables) would cure their disease because they were produced from the earth and contained the healing power of nature.

One 65-year-old Korean woman believed that “Chung-Gook-Chang” had cured her cancer. In fact, Chung-Gook-Chang, the fast-fermented yellow bean paste, was the anticancer food most frequently mentioned by participants:

I ate the powder of dried Chung-Gook-Chang, about 10 kilograms every year. I was unable to eat rice because I had diabetes. So I have had Chung-Gook-Chang at 10 am and 2 pm every day for 6 years, with vegetables. I believe that this powder is responsible for my survival. I don’t eat meat or flour, but I eat greens and yellow bean paste most of the time.

### Coping through Inner Connection with Oneself

#### Seeking Power Inside Oneself

In facing the difficulties brought about by cancer, both Swedish and Korean participants tended to turn to themselves. This is similar to what Fromm (1950) called self-realization. Some people who are struck by a life crisis, such as being diagnosed with cancer, shut their window to the outside and look inside themselves. They may have questions they never considered in the past: “Where did this disease come from? Why do I have to endure this kind of difficulty? Have I done anything wrong? What can I do to change this reality?” As our studies have shown, both Swedish and Korean participants found inner strength that they believed would help them get through the disease.

#### Different Ways of Finding Inner Strength

Although Swedish and Korean participants showed a similarity in coping through an inner connection with oneself, the ways in which they found their inner strength seemed to be different. For Swedish participants, finding inner power meant being spiritually connected with oneself. This did not entail trying to seek a spiritual connection with God or another transcendent power outside oneself. Instead, it was a search for inner spirituality within oneself that they believed would help them cope with cancer.

One 49-year-old Swedish woman who identified herself as a non-theist said:

Religion, no. Spirituality, yes, if it means that you have an inner strength that helps you get through a disease and also faith in the future. Antonovsky’s sense of coherence. Spirituality has helped me find my inner strength, but also why I am who I am and the meaning of my existence.

Whether religious or not, Swedish participants were likely to search for the sacred in themselves when facing a stressful situation. They were able to cope with their illness through realization of the self, which helped them find a spiritual meaning to their life.

In contrast, among Korean participants, coping through an inner connection to oneself seemed to be different from what was prevalent among Swedish participants, which was a spiritual connection with oneself. Korean participants tended to think that they had cancer because they had not taken good care of themselves; they rarely blamed others or the external environment. They believed that their survival depended on how they would cope with the disease. Korean participants showed a considerable level of self-responsibility. Rather than searching for an inner spirituality or spiritual connection with oneself, Korean participants tried to find their own solutions by assuming full responsibility for what had happened to their life. This we can see in the following citation from a 63-year-old Korean man:

You can get as much as you try for. Nothing is for free. A miracle is something you expect to get for free, without any charge. I don’t think anything in life is free of charge. The more you give, the more you get. Give and Take. You should make your effort first and then you will cure your cancer.

### Meaning-Making Coping through Being by Oneself

#### Reaction to Being Alone

During their difficult time struggling with cancer, both Swedish and Korean participants experienced the effects of being alone. However, “being alone” seemed to affect cancer patients in Sweden and South Korea in different ways.

#### Different Interpretation of Solitude

Swedish participants enjoyed being in solitude and appreciated the inner peace it brought. Ahmadi (2006) explained this attitude using the term “positive solitude,” which implies an appreciation of being alone and contemplating one’s own life. Positive solitude was one of the most powerful coping methods for Swedish participants who were able to experience tranquility and relaxation in complete solitude. This experience is different from the feeling of being alone. Feeling alone is a negative feeling caused by not having people around, but experiencing positive solitude has nothing to do with being alone. Positive solitude means enjoying loneliness, and sometimes choosing to be alone despite having the possibility to have people around.

One 34-year-old Swedish woman expressed how much she appreciated being alone:

I liked very much being alone; remaining alone with my thoughts was the most essential way to deal with my problems. I’ve always liked being on my own, writing my thoughts down. Of course, I liked being with other people too, but I liked being alone very much.

On the contrary, Korean participants found something else when they experienced loneliness. Feeling alone with their illness, most Korean participants realized how much they relied on other people and missed being with them. Most Korean participants did not enjoy being alone, but rather longed for gatherings with family or friends while struggling with cancer. They welcomed visits from intimate family and friends and appreciated the care other people offered to them. While they were ill, they seemed to realize the value of having someone around to take care of them, and that they were worth being loved by other people. Some of the Korean participants expressed their gratitude to neighbors who willingly came over and prayed for them. Such feelings of gratitude led them to reflect on whether they had been kind to other people who had difficulties in the past.

One 58-year-old Korean man expressed how much he appreciated other people’s kindness, something he had not expected:

When people visited me and said “You have lived a good life. You will overcome. You will win.” These words gave me great strength. People’s encouragement was of great help. I realized I had a life that was recognized by others. Then I felt relieved.

One 56-year-old Korean woman had been diagnosed with a moderate stage of breast cancer and had to stay in a sterile room from time to time under emergency situations. Nobody had been allowed to enter the room. She had felt lonely in that room and disliked thinking about that time:

There was a window beside my bed. When it was open, I had to see the neon light of a funeral hall. Being alone, I started to cry when I looked at the neon light of funeral hall… Ah…I will be in that funeral hall when I die. I hate those memories of that time. I do hate that moment when I was all alone.

### Praying

In addition to the above-mentioned coping resources, Swedish and Korean participants utilized other diverse methods to seek solace while coping with cancer. For example, Swedish participants enjoyed using spiritual music, healing therapy, and meditation, whereas Korean participants found comfort in praying, meditative calligraphy, or group singing. Here, we see how Swedish and Korean participants’ reasons for praying were considerably different.

Swedish participants sought tranquility through praying. They considered prayer to be a means of achieving relaxation or calmness. The act of praying did not entail pleading to God for a miracle or for direct intercession of the transcendent power to make things better. For Swedish participants, the act of praying was a kind of habit that helped them feel safe and calm. Prayers had almost the same function as meditation had. Another pattern was asking God or a transcendent power to give them strength to fight their illness or to cope with it.

In contrast, for Korean participants, praying entailed desperately pleading to God or a transcendent power. Whether they were religious or not, prayer was the only thing Korean participants could rely on when they were about to enter surgery or undergo treatment. Those who were religious prayed to their God (either Buddha or Jesus Christ), and those who were non-religious prayed to their ancestors or an undesignated transcendent power. Korean participants felt as though they were “grasping at straws” when they prayed. For Korean participants, praying was more than a means of meditation or a way of gaining strength.

One 53-year-old Korean woman relied on praying each time she began treatment:

When I had to go through anti-cancer treatment once a month, I went to a priest and asked him to pray for me. I used to feel much less pain from anti-cancer treatment whenever I said prayers. Sometimes I took that same treatment without saying prayers when my priest was busy on Sunday. Then the treatment was so painful. Maybe it was just my own feeling. But it was much harder without praying.

One 66-year-old Korean man had a strong belief in the healing power of praying. He believed that praying had helped him cope with cancer when he was at his most vulnerable:

A female priest visited me and kept praying for me. She asked me to read the Bible when I had nine days left before surgery. I read the whole bible and got the surgery…After I was discharged from the hospital, I went to church even though it was hard for me to move physically. I still go to church on Sunday and join the choir from 8:30 to 12:30. I would like to join in on the dawn praying so I asked the priest for the real-time video materials. Now I join the praying with my personal computer at dawn (Table 1).

**Table 1:** Results of the Comparative Analysis

Similarities	Differences
Finding Coping Resources in Nature: Seeking comfort in nature	*Sweden* feel spiritually connected with nature
	*Korea* appreciate the value of nature as a remedy for their cancer
Coping through Inner Connection with Oneself: Seeking power inside oneself	*Sweden* search for an inner spirituality within oneself that they believe will help them cope with cancer
	*Korea* find their own solutions by assuming full self-responsibility for what happened in their life
Meaning-Making Coping through Being Alone: Reaction to being alone	*Sweden* enjoy being by themselves in solitude and appreciate inner peace
	*Korea* do not enjoy being alone, but rather long for gathers with family or friends while struggling with cancer
Praying: Seeking comfort in praying	*Sweden* consider prayer to be a means of achieving relaxation or calmness
	*Korea* desperately pleading to God or a transcendent power to survive cancer, whether religious or not

## Discussion

The present study is a part of an international project aimed at exploring meaning-making coping among people who experience cancer in secular societies, and at examining whether culture has any impact on the use of meaning-making coping across the different countries involved. The authors compared the coping methods used by the participating cancer patients in Sweden and Korea, and found that meaning-making coping among cancer patients may be different depending on the culture of the society in which they live. We found four coping methods that Swedish and Korean participants had in common: (1) finding coping resources in nature (seeking comfort in nature); (2) coping through inner connection with oneself (seeking power inside oneself); (3) meaning-making coping through being by oneself; and (4) seeking comfort in praying. Despite the similar patterns of meaning-making coping, participants from Sweden and South Korea showed clear differences within each area of similarity: (1) when seeking comfort in nature, Swedes pursued a spiritual connection with nature, whereas Koreans appreciated the value of nature as remedy to cure their disease; (2) both Swedes and Koreans tried to seek power inside themselves, the former searching for an inner spirituality within oneself and the latter assuming full responsibility and trying to find their own solutions to survive cancer; (3) when experiencing being alone, Swedes tended to enjoy positive solitude and valued inner peace, while Koreans felt afraid of being alone and appreciated their intimate relationships while struggling with cancer; and (4) praying was one of the coping methods used in both Sweden and Korea, but the reason for praying was different. Swedish participants used prayer as a means of experiencing relaxation, while Korean participants (whether they were religious or not) used prayer to desperately plead to a transcendent power to help them survive the difficult situation.

From a cultural perspective, the prominent position of nature in Swedish ways of thinking and Swedish culture might explain why experiences of natural environments play such a central role in coping. Different studies including the European Value System Study (EVSS) have confirmed this (Ahmadi 2006, 2015; Hamberg 1994; Lindén 1994; Uddenberg 1995), all of them indicating that an interest in nature and environmental questions is widespread among Swedes.

The reason Korean participants showed a strong belief in the healing power of nature and anticancer foods from the earth is probably based on their familiarity with the theory of Yin-Yang and the five elements, which is widely shared in East Asian countries (Kim 2013). Yin and Yang indicate two contrasting energies such as the moon and the sun, whereas the five elements indicate the five energies found in fire, water, wood, metal, and earth. According to this theory, Yin-Yang and the five elements also can be applied to human beings, who are also part of nature (Jean 1998). Korean participants tended to believe that the universe consists of those five elements and functions with Yin and Yang in daily living. Therefore, Korean participants’ belief that nature could heal their disease should be understood in the context of the Korean culture and belief system.

Rationalism and the culture of relying on evidence-based information and scientific explanations dominate Swedes’ way of thinking and thus matter in their everyday life (Pettersson and Riis 1994). Swedish society is characterized by a continuous and intensive flow of information and knowledge in relation to, e.g., health issues. Therefore, sets of belief—like Koreans’ belief in the healing power of natural foods and the like—can hardly find fertile soil in Swedish society.

As a way of seeking power inside oneself, Swedish participants searched for an inner spirituality, whereas Korean participants became aware of a high level of self-responsibility. Being responsible for one’s own life and destiny is strongly emphasized in Confucianism, which may explain the mindset of Korean participants, who have been culturally influenced by Confucianism throughout its long history (Pang 1996). Korean participants often mentioned the Chinese character idioms “Chin-Insa-Dae-Chun-Myung” (meaning “Do your best, then God or Higher Power will do the rest”), which originated from the History of Three States in China. They believed that they should do their best to cure their disease and let God (or the Higher Power) do the rest. Here the term God does not necessarily refer to a spiritual or religious being; to Korean people, the word God, which is pronounced *shin* in Korean, may indicate the universe or a transcendent power.^1^

Korean participants in the present study lived in the city of Seoul or in its suburban areas, where the population is more than 25 million. In such a crowded urban society, subways are often jammed and streets are filled with people. Prior to their cancer diagnosis, Korean participants, perhaps like other people in big cities, did not think people mattered as individuals, they were just part of a mass. At this time, Korean participants did not care about how others lived. However, after experiencing a life crisis like cancer, they came to feel lonely and appreciated the kindness that other people showed them. As Wilkes et al. (2003) mentioned, people who have never experienced cancer may find it difficult to have empathy with those who are on “the day-to-day cancer journey” (p. 414), which may have made Korean participants with cancer feel even lonelier. Being alone, Korean participants became more conscious of the value of relationships with close friends and family who encouraged them to cope with cancer.

In contrast, Swedish participants tended to enjoy being alone and sought inner peace through solitude. The Swedes showed a strong tendency toward individualism, whereas the Koreans tended to value togetherness. In the Swedish culture, people are expected to be independent and to manage their own problems themselves. The Korean culture also emphasizes self-responsibility and individuals’ efforts to solve their own problems, but the spirit of “Sahng-Boo-Sahng-Cho” (the four Chinese characters that indicate mutual support)—which means that people should help each other when they have difficulties—is predominant. Because Korea has changed from an agricultural society to an industrialized one, “Doo-Rae” and “Poom-Atssi”^2^ have lost much of their importance, while the spirit of “Sahng-Boo-Sahng-Cho” is still alive among Koreans (Lee 2005). This may explain why people often pray together or bring healthy foods to those who are suffering from cancer.

In our comparative analysis, we found that the reasons for praying differed between Swedes and Koreans. In order to understand the act of praying among Korean participants, we need to look at the Korean culture. Throughout the long history of Korea, people were influenced by Shamanism and were in the habit of praying for mercy whether they were religious or not. Mothers prayed over a bowl of clean water at dawn to plead for health and mercy for their families, and people prayed to an old tree in the village that they believed would guard them from an evil spirit. Still today, parents of high school students who wish to enter good colleges often visit a Church or a Buddhist temple for good fortune. There are numerous stone towers that people have built up on the sides of mountains throughout South Korea. Korean people attach their wishes to a stone and make a tower based on the stones that others have already piled up. Therefore, among Korean people, the act of praying goes beyond a means of meditation or relaxation, which was what Swedish participants used praying to achieve.

In the present study, we found that cancer patients in Sweden were familiar with the construct of spirituality and used the term “spirituality” in their daily communication, whereas their Korean counterparts hardly mentioned “spirituality” in the interviews. However, this does not mean that Korean participants were unconscious of being spiritual. The diversity of meaning-making coping methods clearly showed that Korean participants were familiar with spirituality. They simply did not express it in their language. Although Korean researchers have endeavored to define the construct of spirituality, there is as yet no consensus among the general public. When Korean people hear the term spirituality, they may ask what it means or define it in their own way (e.g., Buddhists would insist that spirituality is from Buddhism, Shamans would say it is beyond existing religions).

South Korea is a collective society where individuals tend to regard being alone as something negative, whereas Sweden is an individualistic society where individuals tend to appreciate being alone and enjoy positive solitude. However, it is interesting to note that although Swedes are individualistic, they sought social altruism; similarly, although Koreans are group oriented, their interests tended to remain at the level of personal healing, although some Korean participants thought about how they could help others in return.

Having learned about the significance of understanding cultural context when trying to investigate meaning-making coping among cancer patients in different countries, the limitations of the present study should be mentioned. First, the study used a qualitative approach and showed that culture is an important factor, but the findings cannot be generalized to all cancer patients in Sweden and South Korea. Future researchers may wish to further investigate diverse aspects of meaning-making coping across countries using either qualitative or quantitative research methods. Second, although the Korean interviews followed the guidelines of the Swedish study and included the same questions, the time gap between the first qualitative study in Sweden and the present one is almost ten years. During this period, there might have been a change in people’s mentality or the social environment in Sweden. Although another quantitative study (Ahmadi 2015) conducted in 2012 verified the results of the earlier qualitative study (Ahmadi 2006), the results of the present study must be interpreted with caution, keeping this limitation in mind.

Despite the limitations, the present study shows that it is important to take cultural differences into consideration when we study meaning-making coping among people who have experienced cancer. Cancer patients use diverse meaning-making coping methods, and these may be spiritual, religious or existential. We would like to conclude that an in-depth understanding of cultural context as well as diverse aspects of meaning-making coping is essential in helping cancer patients survive their disease.

## Footnotes

1 In fact, this idea also exists in Islam, despite the strong position of predestination in Islam and the other Abrahamic religions. Here, we face the theodicy problem and the problem of free will when an omnipotent God is in the picture, but the concept of an omnipotent God is not prevalent in the East Asian religions and ways of thinking.

2 Doo-Rae is a supportive group that residents of a local community organized to help each other with work on the farms during the busy season. While Doo-Rae helped farming, a few members of Doo-Rae played Korean traditional musical instruments such as the Kweng-gwa-ri (a metal gong) or drums to entertain farmers who were exhausted from hard work. Poom-Atssi is similar to Doo-Rae, in that it is also a support system for sharing labor on farms. Compared to Doo-Rae, Poom-Atssi is a small-scale labor exchange, often shared among a few close neighbors regardless of the season.
